# Stenotic Intercondylar Notch as a Risk Factor for Physeal-Sparing ACL Reconstruction Failure: A Case-Control Study

**DOI:** 10.5435/JAAOSGlobal-D-21-00143

**Published:** 2021-07-20

**Authors:** María Tuca, Elizabeth Gausden, Eva Luderowski, Ignacio Valderrama, Tomas Pineda, Hollis Potter, Frank Cordasco, Daniel Green

**Affiliations:** From the Clínica Alemana de Santiago SA—Universidad del Desarrollo (Tuca); The Hospital for Special Surgery (Gausden, Potter, Cordasco, and Green); Johns Hopkins University School of Medicine (Luderowski); Hospital Clínico Mutual de Seguridad (Valderrama); Hospital El Carmen, Santiago de Chile, Chile (Pineda).

## Abstract

Identifying risk factors is crucial for developing strategies that minimize reinjury after anterior cruciate ligament reconstruction (ACLR). This study aims to determine whether certain features of intercondylar notch geometry are associated with failure of physeal-sparing ACLRs in skeletally immature athletes.

**Methods:**

Nine failed physeal-sparing ACLRs were compared with a control subject group of 15 age- and sex-matched intact physeal-sparing ACLRs. Notch width index (NWI), notch angle (NA), and intercondylar notch roof inclination angle (RA) were measured on preoperative MRIs.

**Results:**

Median NWI was smaller in the failed ACLR versus control subject group in coronal (0.23 versus 0.27; *P* < 0.05) and axial planes (0.25 versus 0.27; *P* = 0.055). Median NA was smaller in the failed ACLR versus control subject group in coronal (49.6 versus 61.0°; *P* < 0.05) and axial planes (48.6° versus 54.9°; *P* < 0.05). Median RA was steeper in the failed ACLR versus control subject group (132.0° versus 125.7°; *P* < 0.05).

**Conclusion:**

NWI, NA, and RA were associated with ACLR failure in skeletally immature patients undergoing physeal-sparing reconstruction. A smaller, narrower, and steeper notch may predispose these patients to reinjury.

Anterior cruciate ligament (ACL) injuries are increasingly common in children and adolescents, likely because of an increase in participation in athletic activities coupled with increased recognition of these injuries.^1,2^ According to the Danish Knee Ligament Reconstruction Registry, 6% of all ACL reconstructions are performed in patients younger than 15 years of age.^3^ ACL tears are notable injuries for skeletally immature athletes because they increase the risk of subsequent chondral and meniscal injury.^3,4,5,6^ As a result, the treatment strategy is to perform early ACL reconstruction (ACLR).

Several studies have reported satisfactory objective and subjective outcomes after ACLR in children and adolescents, yet alarmingly, the risk for revision has been estimated as 2.5 to 13× higher in patients younger than 20 years old versus their adult counterparts.^6,7,8,9^ Interestingly, there may be a specific range of pediatric ages that are at greatest risk. In a study investigating the rates of ACLR failure in a pediatric cohort, patients with a median age of 14.3 had a 20% failure rate, whereas similar groups with median ages of 12 and 16.2 experienced failure rates of only 6%.^10^ Despite efforts to optimize surgical treatment, failure of ACL grafts after a successful surgery remains a concern because of the high health and financial burdens. Because revision ACLRs produce inferior results compared with primary ACLRs,^10–12^ prevention is the most effective strategy to avoid reinjury. A thorough understanding of the modifiable (extrinsic) and nonmodifiable (intrinsic) risk factors is crucial to developing strategies to minimize reinjury.

Intrinsic and extrinsic risk factors for tearing a native ACL have been studied extensively. Nonmodifiable or intrinsic risk factors include female sex, increased posterior tibial slope, recurvatum, Beighton score >4, grade 3 pivot, anterior translation >7 mm, ACL size, alignment (fixed valgus), and age (eighth and ninth graders are a particularly high risk group).^10^ Modifiable or extrinsic risk factors include neuromuscular factors (dynamic valgus, quad/hamstring ratios, and gluteus weakness) and the shoe-surface interface.^13,14,15,16^ Several studies have attempted to determine the contribution of intercondylar bony anatomy to the risk of ACL tears, with conflicting results. In 1938, Palmer et al^17^ were the first to suggest that a narrow intercondylar notch may increase the risk of ACL injury because the anatomy causes the ACL to contact the top of the intercondylar notch in full extension and to stretch over the inner margin of the lateral femoral condyle in flexion. In the pediatric population, where growth plates have not yet fused, notch morphology may be an especially important risk factor for ACL tears.^18^

Literature regarding notch geometry has focused predominantly on notch width and notch width index (NWI). Multiple studies support the relationship between narrow intercondylar notch and ACL tears; some reports suggest cutoff values for NWI that indicate increased risk,^18,19,20,21,22^ whereas others have shown no notable correlation between NWI and tear risk.^21,23–25^ The influence of notch geometry on the risk of ACL tears has also been assessed in pediatric populations, where studies suggest that narrow notch sizes are associated with higher risk of ACL rupture.^18,24,26,27^ Importantly, other novel radiographic parameters, such as the intercondylar notch roof inclination angle (RA) and notch angle (NA), have been reported as possible nonmodifiable factors for ACL injuries, with conflicting reports.^28–30^

The aim of this study is to determine whether certain elements of the intercondylar notch geometry are associated with failure of physeal-sparing ACLRs in skeletally immature athletes. Our hypothesis is that a stenotic and steeper notch increases the risk for failure. This is the first study to evaluate notch dimensions as risk factors for physeal-sparing ACLR failure in skeletally immature athletes.

## Methods

**Figure 1:**
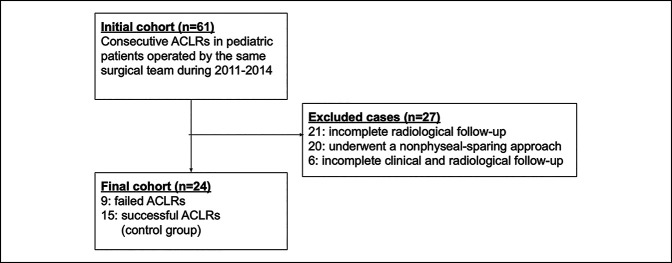
A flow chart showing how failed anterior cruciate ligament reconstruction and control subject groups were selected from the initial cohort.

Nine failed physeal-sparing ACLRs were identified among a cohort of skeletally immature patients treated consecutively by the same surgical team between 2011 and 2014 (Figure 1). For all cases, the team used a retrograde tunnel drilling approach to create an all-epiphyseal femoral socket. All grafts were fixed with cortical buttons in the femur and tibia. All individuals in the cohort followed instructions to abstain from playing sports until at least 12 months after surgery. ACLR reinjury was identified clinically by an attending surgeon and confirmed using MRI. All patients were reconstructed using hamstring autografts, with a median diameter of 9.4 mm for the failed ACLR group (range = 7.5 to 11) and 9.0 mm for the control subject group (range = 7 to 11).

**Figure 2:**
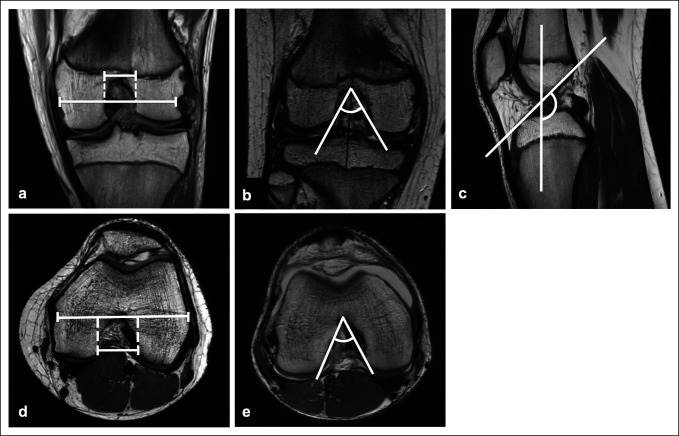
Radiographs showing that the notch width index is the ratio of the intercondylar notch width to the bicondylar width of the distal femur at the level of the popliteus groove in coronal (**A**) and axial (**D**) views. The notch angle is measured at the level of the popliteus groove, tracing the opening of the intercondylar notch in coronal (**B**) and axial (**E**) views. Roof inclination angle is the obtuse angle formed by the intersection of a line over the Blumensaat line and a line parallel to the long axis of the knee in the midsagittal view (**C**).

Failures were compared with an age- and sex-matched control subject group of 15 intact physeal-sparing ACLRs from the same initial cohort who had returned to athletics with a minimum of 2 years clinical and radiological follow-up (Figure 1). NWI and NA were measured in coronal and axial proton density-weighted MRI studies using a previously reported method.^31,32^ The intercondylar notch width was measured on the coronal and axial MRI slices that best showed the popliteus groove. NWI is the ratio of the notch width to the bicondylar width on the same image slice (Figure 2, A and D). NA was also measured on the same coronal and axial images (Figure 2, B and E). RA was measured on a midsagittal proton density-weighted MRI images and defined as the obtuse angle between a line parallel to the long axis of the knee and a line parallel to the intercondylar roof or Blumensaat line (Figure 2C). Measurements were made by a single physician researcher. Chi-squared and Wilcoxon tests with an alpha level of 0.05 were used for statistical analysis (STATA software, version 14, StataCorp LP).

## Results

Both study groups had comparable distributions of age and sex (Table 1). Median coronal NWI in the failed ACLR group (0.23; range = 0.16 to 0.27) was markedly smaller than in the control subject group (0.27; range = 0.20 to 0.31). In the axial plane, although the failed ACLR group NWI (0.25; range = 0.20 to 0.29) was also smaller than in the control subject group (0.27; range = 0.21 to 0.33), the association was not statistically significant (*P* value = 0.055). Median NA in the failed ACLR group was markedly smaller than in the control subject group in both the coronal (49.6°; range = 35.3° to 57.9° versus 61.0°; range = 43.8° to 71.9°) and axial planes (48.6°; range = 37.4° to 55.3° versus 54.9°; range = 41.9° to 71.9°). Median inclination of the RA was markedly steeper in the failed ACLR group (132.0°; range 147.1° to 123.4°) compared with the control subject group (125.7°; 146.8° to 113.9°) (Table 2).

**Table 1:** Demographic Characteristics Comparison Between Failed ACLR Group and Control Group

	Failed ACLR Group (n = 9)	Control Subject Group (n = 15)	*P*
Chronological age, median (range)	14.9 (13.3-17.1)	14.0 (12.4-16.7)	0.107
Bone age, median (range)	14.0 (12.0-15.0)	13.5 (12.0-16.6)	0.264
Male: female	7: 2	12: 3	0.897

ACLR = anterior cruciate ligament reconstruction

**Table 2:** MRI Knee Measurement Comparison Between Failed ACLR Group and Control Group^a^

	Failed ACLR Group	Control Subject Group	*P*
Coronal NWI	0.23 (0.16-0.27)	0.27 (0.20-0.31)	0.017
Axial NWI	0.25 (0.20-0.29)	0.27 (0.21-0.33)	0.055
Coronal NA	49.6° (35.3°-57.9°)	61.0° (43.8°-71.9°)	0.008
Axial NA	48.6° (37.4°-55.3°)	54.9° (41.9°-71.9°)	0.036
RA	132.0° (147.1°-123.4°)	125.7° (146.8°-113.9°)	0.021

ACLR = anterior cruciate ligament reconstruction, NWI = notch width index, NA = notch angle, RA = notch roof angle

a Data shown as median (range).

## Discussion

All three measurements used to assess intercondylar notch morphology (NWI, NA, RA) were markedly associated with ACLR failure, suggesting that in patients undergoing a physeal-sparing ACLR and a narrow and steeper notch predisposes to reinjury. Most of the literature studying notch geometry and ACL tears has focused on native ACL injuries in adult populations. This is the first study that evaluates intercondylar notch morphology as a risk factor for ACLR failure in a pediatric cohort that underwent physeal-sparing surgery.

Reinjury after ACLR in pediatric patients is associated with high morbidity, inferior long-term outcomes, and a long and costly rehabilitation. Importantly, patients younger than 20 years old are far more likely to experience ACLR failure than their adult counterparts, and eighth and ninth graders are at particularly high risk.^7,10^ As a result, the identification of risk factors in this population and subsequent application of preventive measures is critical. A common theory regarding the high rate of reinjury in teenagers was that young athletes were encouraged to return to contact sports prematurely, but according to the Danish Knee Ligament Reconstruction Registry, a large proportion of failures occurred after the first year of recovery, which is the recommended time for abstaining from athletics after ACLR surgery.^3^

Most evidence indicates that a narrow notch may put the ACL at a greater risk of initial injury. A meta-analysis by Zeng et al^33^ concluded that a lower NWI or notch width stenosis predisposes an individual to ACL injury. This association has also been reported in pediatric populations, concordant with the findings of this study.^18,21,24^ Domzalski et al^17^ reviewed 46 MRIs of children and adolescents with torn ACLs and compared them with 44 healthy control subjects. Similar to this study, they found that the NWI was markedly larger in intact knees (0.27) versus joints with torn ACLs (0.24). Swami et al^26^ compared three-dimensional notch volumes in 50 MRI studies of pediatric patients with torn ACLs versus healthy control subjects and showed that three-dimensional notch volume was markedly smaller in knees with ACL tears than in intact knees. These data correspond with our finding that smaller notch width, albeit a 2-dimensional feature, correlates with ACLR failure and support the conclusion that less space in the notch may be a risk factor for reinjury.

Some authors have attempted to set cutoff NWI values that define notch stenosis. Domzalski et al,^18^ in their pediatric MRI-based study, proposed an average NWI of 0.24 as a risk factor for ACL tears. Souryal et al^25^ determined that a NWI of 0.231 ± 0.044 should be considered a normal intercondylar notch ratio and that values < 0.2 represent risk for ACL injury. Other reports have established the cutoff value for NWI as 0.25 or 0.21, ranges that would be reasonable given the findings of this study.^20,34^

The method for measuring notch width is controversial. Although plain radiographs are fast and less expensive to obtain than MRI, variability in the technique and magnification make them less accurate than MRI measurements.^31,35^ In addition, some authors suggest that absolute width rather than NWI is the best way to quantify notch shape because bicondylar size might not increase in proportion to height.^36^ Further studies are needed to identify the best tool for determining the notch geometry that is most informative for determining reinjury risk.

Some authors have proposed that a narrow notch may predispose patients to ACL tears because of impingement of the graft or native ligament in the lateral femoral condyle or intercondylar roof during knee extension and internal rotation.^32,37^ Anatomic studies have reported that smaller intercondylar notches house correspondingly smaller and weaker ligaments with an increased risk of injury.^18^ Although the efficacy of notchplasty is still controversial, proponents suggest that if a narrow notch is noted intraoperatively during an ACL repair, a notchplasty should be performed with the objective of widening the notch and avoiding impingement of the graft.^33^

The contribution of intercondylar NA to ACL reinjury risk has been less studied. The impetus for measuring the opening angle of the notch is that the size of the angle may be an important feature of notch morphology.^15^ For example, in cases where notch shape is wide deeply but narrow at the base, the NA may be abnormal despite a normal NWI. A smaller NA may predispose to ACL tears because anterior translation forces and knee valgus may lead to impingement.^26^ Currently, no consensus about the role of NA in predicting ACLR failure risk exists. Some authors suggest that angles < 50° increase the risk of ACL injury,^29,35^ whereas others do not report a notable association.^28^ The results of this study support the potential role of NA as a risk factor because the average NA of the failed ACLR group was fewer than 50°, whereas the control subject group average was above that cutoff. Further studies are needed to establish whether an association between a smaller NA and ACL injuries exists.

The inclination of the intercondylar notch roof angle is a novel radiographic parameter that describes the orientation of the notch in relation to the distal femoral diaphysis. Some studies have evaluated this metric, but using varied and nonstandardized methodologies, which makes comparison difficult. The orientation of the roof angle might predispose to graft failure by impinging the tissue or by leading to misplacement of the femoral tunnels.^30^ RA, measured as previously described, ranges from 135° to 147° in reports based on plain radiographs, MRIs, and cryosectional anatomic studies.^38^ The results in our study likely differ from previously reported RA values because RA is a relatively new parameter, and there is not yet a consensus regarding how to best measure it. Some publications use a line parallel to the posterior femoral cortex, whereas other studies, such as ours, use the long axis of the knee. Extension lag in injured knees might also affect our measurements of RA. Further studies are needed to standardize how to measure the RA and test the validity and reproducibility of this metric.

This study has several limitations. Many patients were excluded from the study because they had fewer than two years of follow-up or had two years of clinical follow-up but lacked follow-up imaging, which limited the size of the cohort. Additional patients were excluded because they did not undergo a physeal-sparing surgical approach. Moreover, measurements were performed by one observer, which did not allow reliability testing, although NWI and NA have been validated previously in pediatric patients.^29,39,40^ In addition, the validity and reproducibility of roof angle parameters have not yet been evaluated. Importantly, although a larger sample size would increase the power of the study, the sample used in this study was sufficient to generate statistically significant results while restricting the injured cohort to age- and sex-matched comparisons.

## Conclusion

This study is the first to suggest that NWI and NA may be predictors for physeal-sparing ACLR failure in skeletally immature athletes. A steeper inclination of the notch roof was also related to ACLR failure in this population, possibly because of altered biomechanics of the reconstructed ACL.
